# Expression of Cancer/Testis genes in ductal carcinoma in situ and benign lesions of the breast

**DOI:** 10.18632/oncoscience.4

**Published:** 2013-12-12

**Authors:** Otavia L. Caballero, Sami Shousha, Qi Zhao, Andrew J.G. Simpson, R. Charles Coombes, A. Munro Neville

**Affiliations:** ^1^ Ludwig Collaborative Laboratory, Ludwig Institute for Cancer Research, Department of Neurosurgery, The Johns Hopkins University School of Medicine, Baltimore, MD USA; ^2^ Imperial College Healthcare NHS Trust & Imperial College, London, Charing Cross Hospital; ^3^ Ludwig Institute for Cancer Research, New York, NY., USA; ^4^ Current affiliation: Orygen Biotecnologia, São Paulo, Brazil.

**Keywords:** cancer/testis genes, DCIS, ER negative

## Abstract

Cancer/testis (CT) genes represent a unique class of genes, which are expressed by germ cells, normally silenced in somatic cells, but activated in various cancers. CT proteins can elicit spontaneous immune responses in cancer patients and this feature makes them attractive targets for immunotherapy-based approaches. We have previously reported that CTs are relatively commonly expressed in estrogen receptor (ER) negative, high risk carcinomas. In this study, we examined the expression of selected CT genes in ductal carcinoma in situ (DCIS), lobular carcinoma in situ (LCIS) and benign proliferative lesions of the breast. ER negative DCIS were found to be associated with significant CT gene expression together with HER2 positivity and a marked stromal immune response.

## INTRODUCTION

Cancer/testis (CT) genes, normally expressed only in the testis at different stages of sperm development, become activated in various malignancies [1, 2]. While over 150 CT antigens have been isolated and characterized [3], they remain relatively unexplored in both the clinical and laboratory context. The expression of CT genes varies greatly between tumor types being most frequent in melanomas, bladder, hepatic and lung carcinomas [2]. Breast cancer has been regarded as relatively CT-poor. We have found, however, that breast cancer is not uniformly CT-poor; CTs are relatively commonly expressed in estrogen receptor (ER) negative, high risk carcinomas [4, 5]. In this form of breast cancer MAGEA3, for example, is expressed at 15-26% [4, 6, 7] as compared with around 6% in unselected breast cancers. Because of the limited therapeutic options for ER-negative breast cancers, vaccines based on CT-X antigens might prove to be useful [4].

The expression of CT genes in intraductal proliferative lesions of the breast has been poorly investigated. Ductal carcinoma in situ (DCIS) now supported by a great deal of genetic and molecular cytogenetic evidence [8] is considered the direct precursor lesion for invasive breast cancer (IBC). Pre-invasive DCIS is associated with excellent 5-year survival rates, however, it is estimated that at least one-third of the lesions progress to IBC [9]. Identification of those DCIS which are more prone to progress to overt cancer remains difficult to discern although steroid receptor negativity and the presence of HER have been proposed as possible indicators [10, 11]. Our aim in this study was to ascertain if there was a group of DCIS/LCIS that were ER negative and in which CT expression might occur and which therefore would represent possible therapeutic targets for immunotherapy.

## RESULTS

### Analysis of CT gene expression in a publicly available DCIS microarray dataset

We interrogated a publicly available microarray dataset [14] for the expression of 45 probesets corresponding to 42 testis-restricted CT genes (Supplementary Table 1) in 31 pure DCIS samples. We first compared the expression of each probeset between the DCIS samples and six normal mammary tissues. We found that seven CT genes (CT45A1, CT45A5, CT47A1, PLAC1, SSX2, SSX4B and SYCP1) were significantly overexpressed in DCIS samples compared to normal mammary tissue (Supplementary figure 1). Additionally, we analyzed the differential expression of CT genes between ER positive and ER negative and high grade and low-grade DCIS. NY-ESO-1, CT46, CXorf61 and LEMD1 were found to be significantly overexpressed in ER negative compared to ER positive DCIS (Supplementary figure 2). From all CT genes analyzed, NY-ESO-1 was the only one found to be overexpressed in grade 3 DCIS compared to grades 1 and 2 (P=0.0432). Moreover, we evaluated CT gene expression according to DCIS subtypes. We have previously shown that CT-X genes are more frequently expressed in the basal subtype [4]. Due to the small number of DCIS cases in this dataset, for statistical purposes, we dichotomized the samples into two categories: basal and non-basal subtypes, the latter included luminal A, luminal B, HER2 and normal-like subtypes. We found that CT46, CXorf61 and LEMD1 are significantly overexpressed in samples of the basal subtype compared to the non-basal subtypes (Supplementary figure 3). One DCIS sample (DCIS-142) expressed very high levels of seven CT genes (NY-ESO-1, CT46, LEMD1, CXorf61, CT47A1, MAGEA1 and MAGEA10) as compared to the mean expression levels of these CTs in the normal breast samples (Supplementary figure 4). This is consistent with the coordinated expression of CT genes described in invasive tumors [15]. Interestingly, DCIS-142 is a hormone receptor negative, high grade, basal subtype DCIS sample.

### Expression of CT genes in breast tissues

To validate and extend our findings from the in silico analyses, we evaluated CT gene expression in RNAs from FFPE tissues prepared from 23 DCIS cases (Table 1), 11 of which presented HER2 overexpression and seven were ER negative. In addition to the DCIS samples, we have also analyzed other benign proliferative and premalignant breast lesions such as atypia, hyperplasia and LCIS (detailed in Table 1). Among all 40 samples tested, only one (sample # 25) did not yield amplifiable RNA. From the list of CT genes that were found to be differentially expressed between the subsets of DCIS analyzed in silico, we selected the ones for which adequate primer pairs could be designed for evaluating RNA expression from FFPE tissues. We investigated the expression of CT46/HORMAD1, NY-ESO-1, CXorf61, LEMD1, PLAC1, CT45A1 and CT47A1 by RT-PCR. We decided to also include MAGEA3 in this analysis as it is the CT gene that is currently in the most advanced stage in clinical trials of therapeutic CT-based cancer vaccines [16]. NY-ESO-1 mRNA expression was found in 13/23 DCIS samples (56.5%), 1/5 LCIS (20%) and in 6/12 benign proliferative lesions (50%). MAGEA3 and CXorf61 expression was found in 3/23 (13%) of the DCIS samples but neither gene was detected in LCIS or benign lesions. CT46 expression was found in 7/23 (30.4%) and LEMD1 in 8/23 (34.8%) DCIS samples and in 1/12 (8.3%) and 3/12 (25%) benign lesions, respectively. CT45A1, CT47A1 and PLAC1 were positive in one DCIS sample each. Eleven of the 23 DCIS samples analyzed by RT-PCR were found to express two or more CT genes.

**Table 1:** Summary of pathological type and markers analyzed in this study

						RNA					IHC		
Patient	Histology	Grade#	Ki67&	HER*	ER*	CT46*	ESO*	MAGEA3*	CXORF61*	LEMD1*	CD8	ESO*	MAGEA*
1	DCIS	1	1	0	1	0	0	0	0	1	0	0	0
2	DCIS	1	2	0	1	0	0	0	0	0	1 (focal)	0	0
3	DCIS	3	1	1	1	0	0	0	1	0	4 (focal)	1	0
4	DCIS	3	1	1	0	1	1	0	1	1	4	1	0
5	DCIS	2	1	0	0	0	1	1	0	0	4	1	1
6	DCIS	2	2	0	1	1	1	0	0	0	1 (focal)	1	0
7	DCIS	2	1	0	1	0	1	0	0	0	3 (focal)	1	0
8	DCIS	2	1	1	1	0	1	0	0	1	4 (focal)	0	0
9	DCIS	1	2	0	1	0	0	0	0	1	0	1	0
10	DCIS	1	1	0	1	1	1	0	0	1	3 (focal)	0	0
11	DCIS	3	2	1	0	1	1	1	0	0	4	1	1
12	DCIS	1	1	0	1	0	1	0	0	0	0	1	0
13	DCIS	2	1	0	1	0	0	0	0	0	0	1	0
14	DCIS	3	3	1	1	0	0	0	0	0	4	1	0
15	DCIS	1	1	1	1	1	0	0	0	0	0	1	0
21	DCIS	1	1	1	1	0	0	0	0	1	4 (focal)	1	0
27	DCIS	2	2	1	0	1	1	0	0	1	0	1	0
28	DCIS	3	2	1	0	0	0	0	0	0	4	1	0
29	DCIS	1	0	0	1	0	0	0	0	0	0	1	0
30	DCIS	3	3	1	0	0	1	1	0	0	4	1	1
32	DCIS	2	1	1	0	1	1	0	1	0	0	1	0
33	DCIS	1	1	0	1	0	1	0	0	1	0	1	0
35	DCIS, florid atypia	NA	2	0	1	0	1	0	0	0	1	1	0
16	LCIS	NA	0	0	1	0	1	0	0	1	1	0	0
17	LCIS	NA	0	0	1	0	0	0	0	0	0	0	0
18	LCIS	NA	0	0	1	0	0	0	0	0	1 (focal)	0	0
19	LCIS	NA	0	0	1	0	0	0	0	0	4	0	0
20	LCIS	NA	0	0	1	0	0	0	0	0	1	0	0
22	flat atypical hyperplasia	NA	0	0	1	0	0	0	0	0	0	0	0
23	radial scar;florid	NA	1	0	1	0	0	0	0	0	3 (focal)	0	0
24	Benign	NA	1	0	1	1	1	0	0	0	0	1	0
31	radial scar	NA	0	0	1	0	0	0	0	0	0	1	0
36	radial scar; florid adenosis	NA	1	0	1	0	0	0	0	0	0	1	0
37	radial scar	NA	3	0	1	0	1	0	0	1	0	1	0
34	cystic apocrine	NA	1	1	1	0	1	0	0	1	0	1	0
25	mild adenosis	NA	2	0	1	NA	NA	NA	NA	NA	0	0	0
26	Intraductal papiloma	NA	1	0	1	0	0	0	0	1	4	1	0
38	Flat adenosis	NA	1	0	1	0	1	0	0	1	0	1	0
39	focal Hyperadenosis	NA	1	0	1	0	1	0	0	0	0	0	0
40	radial scar adenosis	NA	1	0	1	0	1	0	0	0	0	0	0

* 0=negative; 1= positive

&Ki67: 0=negative; 1= minimum; 2=moderate; 3=marked

### Expression of CT protein in breast tissues

**Figure 1:**
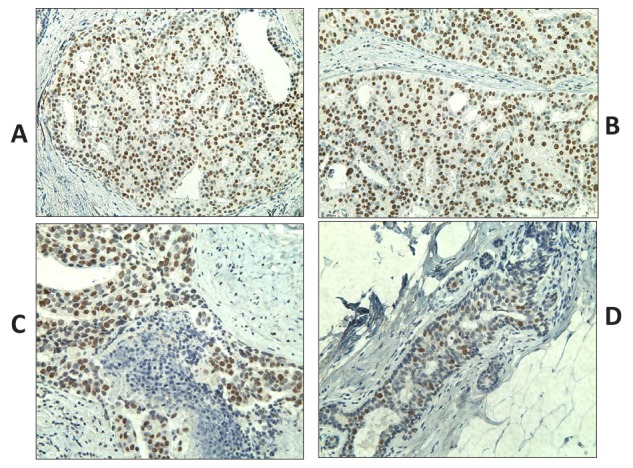
Immunohistochemistry staining of DCIS samples using monoclonal antibody specific to NY-ESO-1 (clone E978) (shown in brown) Sections presented variable cytoplasmic and nuclear NY-ESO-1 staining, typically showing either focal and scattered positive cells (A and B) or intense and diffuse positivity (C and D) in >90% of tumor cells. Original magnification, ×200.

**Figure 2:**
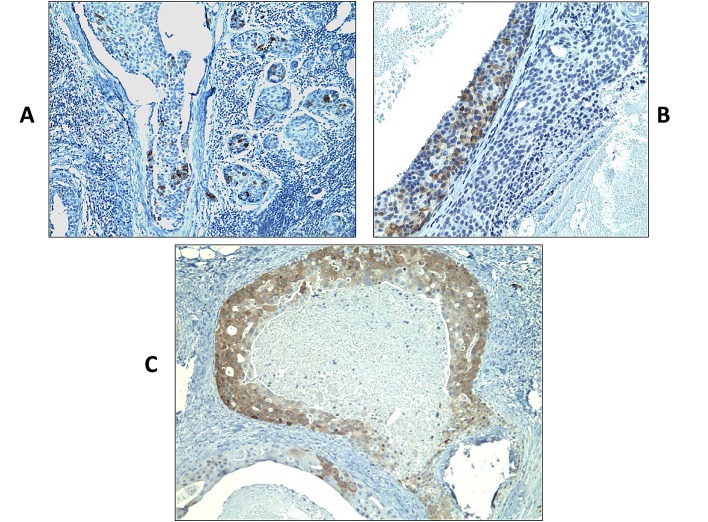
Immunohistochemistry staining of DCIS samples using monoclonal antibody specific to MAGEA (clone 6C1) (shown in brown) Sections presented variable cytoplasmic MAGEA staining, typically showing either focal and scattered positive cells (A and B) or intense and diffuse positivity (C) in >90% of tumor cells. Original magnification, ×200.

Based on the encouraging RNA expression results, we decided to analyze further the expression of CT antigens by IHC using previously characterized antibodies specific to MAGEA and NY-ESO-1 in the same samples (Table 1). Adequate antibodies were not available for analyzing the expression of the remaining CT proteins investigated in this study. Similar to the results of the RT-PCR, NY-ESO-1 protein expression was detected not only in DCIS but also in benign proliferative lesions whereas areas of normal breast were always negative for NY-ESO-1 expression (in 19/23 or 82.6% of DCIS; and in 7/12 or 58.3% of benign proliferative lesions), but it was not detected any LCIS tested. In some cases, NY-ESO-1 was found to be diffusely and homogenously detected in almost all tumor cells (Figure 1A, B and C) and in others, NY-ESO-1 expression was heterogeneous. Some cases showed patchy expression, whilst others showed only small clusters of tumor cells with strong expression within a background of CT-negative tumor cells (Figure 1D). NY-ESO-1 was more frequently detected in the nuclei but combined nuclear and cytoplasmic or purely cytoplasmic staining was also observed (Figure 1). RT-PCR and IHC results were concordant in 56.4% of the cases. Six cases (15.4%) were negative by IHC but positive by RT-PCR, which could be explained by the lower sensitivity of IHC versus RT-PCR. Conversely, 11 cases (28.2%) were positive by IHC but negative by RT-PCR. A possible reason for this is mRNA degradation due to the formalin fixation process and contamination with RNAses or because only a small cluster of tumor cells with strong expression was seen amongst a background of CT-negative tumor and normal cells within the section. MAGEA protein was observed in three DCIS cases, the same cases where MAGEA3 mRNA expression was detected. Similarly to NY-ESO-1, and except for one case, the positivity was observed in a small cluster of tumor cells with strong expression amongst a background of CT-negative tumor cells (Figure 2).

### ER negative DCIS

Analysis of the present data disclose a group of ER negative DCIS for whom CT expression and other characteristics are highly akin to those found in overt breast cancer, namely the more frequent occurrence of CT expression in the absence of ER (Table 2). MAGEA3 is only expressed in ER negative DCIS. Moreover, 6 of the 11 HER2 positive DCIS were found to be ER negative. There was a significant correlation of HER2 positivity with marked (4+) CD8+ lymphocytic infiltration (P=0.0075). A marginally significant association of MAGEA positivity with high CD8+ lymphocytic infiltration was also observed (P=0.0545).

**Table 2:** Correlation between CT expression or HER2 presence and ER status

No of Cases		ER positive	ER negative	P-value
16	7	
NY-ESO-1*	present	7	7	0.0858
	absent	8	1	
MAGE A3*	present	0	3	0.0198
	absent	16	4	
CT46*	present	3	4	0.1374
	absent	13	3	
HER2**	present	5	6	0.0272
	absent	11	1	

* RT-PCR results

** IHC results

## DISCUSSION

Currently, it is difficult to predict which patients with DCIS will progress to invasive breast cancer. If we were able to do this, it would have important implications for the treatment of patients with DCIS. In view of these difficulties several studies have resorted to the use of various biomarkers in an attempt to provide better histological and prognostic diagnoses. HER2 and HER2 in association with Ki67 expression have been reported to be correlated with an increased tendency of DCIS to recur [17-19]. For example, 34% of HER2 DCIS has been reported as having a higher histological grade and to be of the comedo type. In addition, some cases of HER2 positive ER negative DCIS have been noted to be associated with a higher rate of subsequent DCIS recurrence but not with later invasive cancer [17].

Our previous work has highlighted ER negative mainly but also triple negative breast cancers as those lesions most associated with the expression of CT genes such as MAGEA and NY-ESO-1 [4, 7]. This led us to examine the early proliferative breast lesions to ascertain the incidence of CT expression. When we interrogated a publicly available DCIS microarray dataset [14], we found several CT genes to be significantly enriched in hormone receptor negative, high grade and basal subtype DCIS. The analysis of an independent set of breast lesions in the present study at both RNA and protein levels has revealed that benign lesions and DCIS may express NY-ESO and/or other CT genes (Table 1) but only DCIS expressed MAGEA was shown to be significantly associated with ER negativity. Eleven of the DCIS of the present small series were HER2 positive. Of potential greater interest are the six cases that are HER2 positive and ER negative. These lesions fall into the molecular classification of HER2 positive cases and basal cancers as opposed to the remainder which are of luminal type A and B. Recurrences of DCIS have been noted by others in each of these categories [17, 20].

As noted previously, CTs represent a unique class of tumor antigens, which are expressed by germ cells, normally silenced in somatic cells, but activated in a wide variety of cancer types [1, 2]. Although they are not unique to cancer cells as is illustrated here, but rather shared with germ line cells, they have been shown to be capable of eliciting cellular and/or humoral immune responses what makes them ideal antigens for cancer immunotherapy [2].

It is now recognized that incidence of CT gene expression is higher in ER negative and triple negative breast tumors [4, 7, 21]. Breast cancer screening programs aimed at earlier detection, hopefully at the pre-invasive stage, have detected a variety of conditions some of which are known to carry a 2 to 10-fold increased chance of progressing to overt cancers [22] including DCIS and LCIS. In the present study, we have uncovered a subset of DCIS that are ER negative and CT positive and which therefore may highlight a group which on further study may be more prone to recur and therefore become candidates for immunotherapeutic approaches to avoid progression into invasive breast cancer. Interestingly, in a recent study, tumor expression of HER2 and estrogen receptor negativity predicted clinical response and complete pathologic response, respectively, to HER2-pulsed DC1 vaccines [23]. This may suggest that the ER negative subset of DCIS in which the CT genes are frequently expressed could be more susceptible to immunotherapeutic approaches.

NY-ESO-1 immunostaining in our study reveals a high frequency of NY-ESO-1 protein expression in both DCIS and benign lesions. This frequency is actually much higher than that determined by RT-PCR, and the discrepancy is probably due to the heterogeneous and focal patterns of NY-ESO-1 expression. This staining pattern may be correlated with the proliferative nature of these lesions and was demonstrated before in breast fibroadenomas [24]. Although NY-ESO-1 is a highly immunogenic tumor antigen, its presence in benign lesions would not make it a useful target for immunotherapeutic approaches for cancer treatment. Of more potential clinical interest is the finding that only seven of the 23 DCIS express CT46 and of these, two cases also show a marked presence of CD8+ TILs. Similarly, only three out of the 23 DCIS express MAGEA3, and in all of them a marked CD8+ infiltration could be observed. Because the expression of these two CTs in invasive breast cancer is correlated with more aggressive disease [5, 7], we could speculate that they might define a group of DCIS which is more likely to proceed to invasive breast cancer. However, due to our finding that these lesions are accompanied by sometimes heavy CD8+ lymphocytic infiltrates, it may be that the lesions that are highly immunogenic due to the expression of CT genes are the ones that are unlikely to progress. To clarify this issue, it would be necessary to examine a larger series of DCIS cases that over a 10-15 year follow-up period to ascertain whether recurrences have or have not occurred. In such a series, it will be important to add other biomarker indices that might be of value in determining the outcome, including but not limited to PADI2 [25], FGFR1[26], SOX2 [27], EZH2 and ALDH1 [28], all of which in other unrelated studies have been proposed to play a role in progression of early breast lesions to overt cancer.

In conclusion, our novel findings that CT genes are expressed in premalignant lesions of the breast represent an entry point to future work focused on the investigation of the value of CT gene expression as a biomarker of progression and/or as therapeutic targets for immunotherapeutic approaches aimed at preventing the progression of these lesions.

## MATERIAL AND METHODS

### Breast cancer microarray dataset processing

The transcription profile of CT genes present in GSE26304 was obtained from NCBI GEO database (http://www.ncbi.nlm.nih.gov/geo/) which is based on Agilent Whole Human Genome Oligo Microarrays 44k (Supplementary Table 1). Data from 31 pure DCIS and six normal breast tissue samples, included as controls, were downloaded. Pathological data available included ER and HER2 status, grade and molecular subtype (Supplementary Table 2).

### Patients and samples

We obtained operative core biopsy material, from female patients, selected to represent a wide spectrum of benign and atypical lesions as well as cases of DCIS of various grades and LCIS (Table 1). Clinical samples were de-identified and obtained without individual consent under a protocol approved by the Charing Cross Hospital Institutional Review Board. All tissues were processed routinely by fixation in 10% neutral formalin for 24-48 hours and embedded in paraffin wax. Pathology data were obtained from the pathology reports and histological re-evaluation of slides.

### Reverse transcription-PCR

Total RNA was extracted from 3 ×10 μm formalin-fixed paraffin embedded (FFPE) sections using the RNeasy FFPE Kit. cDNA was prepared with the Omniscrip RT Kit (Qiagen) using 1 microgram of total RNA. PCR primers, optimized for use on formalin-fixed, paraffin embedded (FFPE) material, were used for the detection of CT46, NY-ESO-1, MAGEA3, LEMD1, CXORF61, PLAC1, CT45A1, CT47A1 and TBP, which was used as an endogenous control gene in all samples. RNA from a FFPE testis sample was used as positive control and negative control without cDNA was also included in all reactions. Primer sequences and expected amplicon sizes are listed in Supplementary Table 3. JumpStart REDTaq ReadyMix PCR Reaction Mix (Sigma, St. Louis, MO), was used for PCR amplifications after the addition of 5 pmoles of each primer and one μl of the cDNA solution in 25 μl final volume. The PCR conditions were 95°C for 3 minutes followed by 42 cycles at 95°C for 15 seconds and 60°C for 30 seconds and 72°C for 30 seconds, followed by a final 7-min extension. PCR products were visualized by UV illumination of 2% ethidium bromide stained agarose gels. The identity of the amplicons was verified by Sanger sequencing of the PCR product obtained using the testis sample.

### Immunohistochemistry

Archival H&E-stained slides of all cases were reviewed and a representative section was selected from each case. Several new 5-micron sections were cut from each selected paraffin block. One section was stained by H&E, to ensure the presence of target lesion. The others were used for immunohistochemistry (IHC) using the immunoperoxidase technique as follows: For ER (clone SP1), PgR (1E2) and HER2 (4B5) Roche Ventana Benchmark XT auto-stainer was used. All antibodies were supplied pre-diluted and a 30 minute step of antigen retrieval in citrate buffer was used. For Ki67, MM1 mouse monoclonal antibody diluted 1/100 was used after 30 minutes antigen retrieval in ER1 (citrate) using Leica Bond Autostainer. NY-ESO-1 (clone E978) and MAGEA (clone 6C1) were detected by IHC using previously validated and described reagents and methods [12, 13]. Infiltrating CD8+ cells, demonstrated by IHC using C8/144B antibody, were evaluated by two pathologists (SS and AMN) who were blinded to the clinical characteristics and outcomes of the patients. The categories utilized were: negative, when no lymphocytic infiltrate was found within the tumor; and 1+ to 4+ according to the intensity of the infiltrate. For statistical purposes cases with no infiltrate or ≤ 1+ were classed as “low” and the remainder, 2+ and above, as “high”.

### Statistical analysis

Statistical analyses were performed with SPSS version 20.0 (SPSS Inc, Chicago, IL, USA) and with GraphPad Prism 5. Differences between specific patient groups based on clinicopathological characteristics were determined using Fisher's exact test. A two-tailed P < 0.05 was considered statistically significant.

## SUPPLEMENTARY FIGURES AND TABLES








