# Supplementary material for: Expression of Cancer/Testis genes in ductal carcinoma in situ and benign lesions of the breast
Source: Oncoscience. 2013 Dec 12;1(1):14–20. doi: 10.18632/oncoscience.4 (PMC4295763; doi:10.18632/oncoscience.4)
Supplement: Supplementary file 1 [file oncoscience-01-0014-s001.pdf]

Expression of Cancer/Testis genes in ductal carcinoma in situ and benign lesions of the breast- Caballero et al

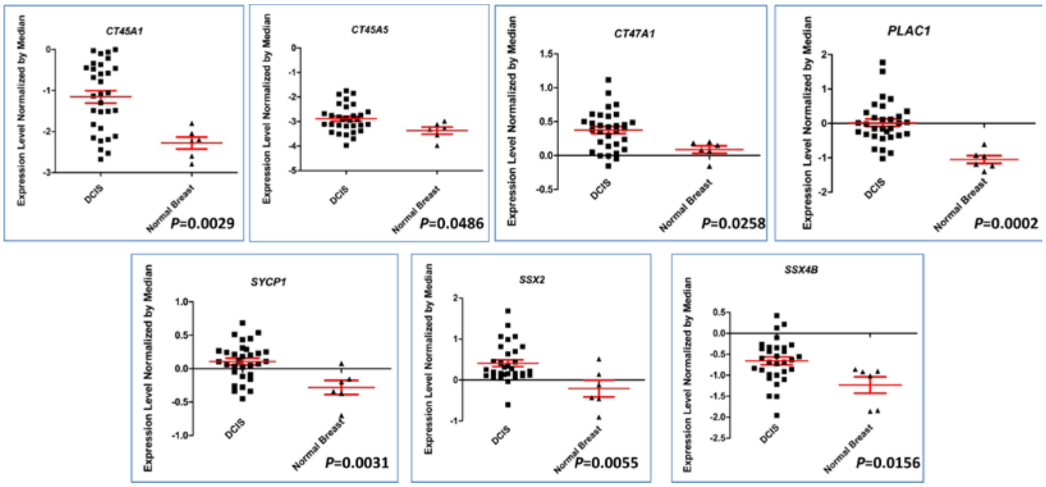

Supplementary Figure 1: Levels of *CT45A1*, *CT45A5*, *CT47A1*, *PLAC1*, *SYCP1*, *SSX2 and SSX4B* in normal mammary tissue (n=6) and DCIS samples (n=31) in the GSE26304 dataset. In red are the means ± SEM.

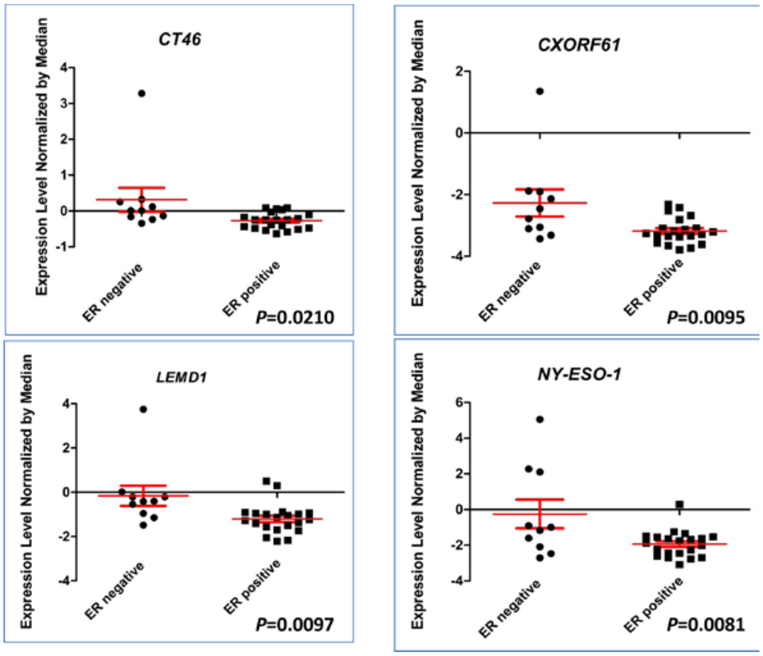

Supplementary Figure 2: Levels of NY-*ESO-1*, *CT46*, *CXorf61* and *LEMD1* in ER negative (n=10) and ER positive (n=21) DCIS samples in the GSE26304 dataset. In red are the means ± SEM.

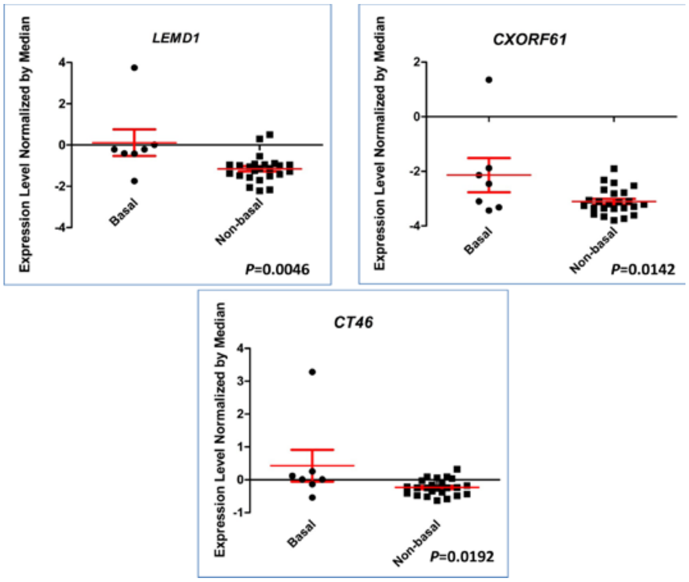

Supplementary Figure 3: Levels of *LEMD1*, *CXorf61* and *CT46* in basal (n=7) and non-basal (n=24) DCIS samples in the GSE26304 dataset. In red are the means ± SEM.

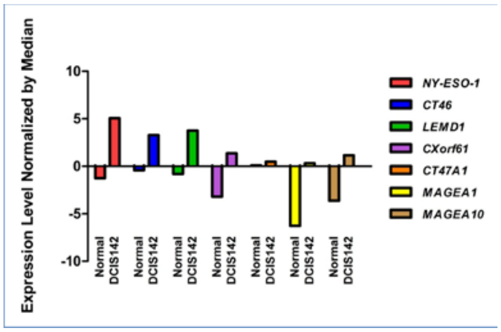

Supplementary Figure 4: Expression levels of NY-*ESO-1*, *CT46*, *CXorf61*, *LEMD1*, *CT47A1*, *MAGEA1* and *MAGEA10* in sample DCIS-142 from the GSE26304 dataset compared to the mean levels in six normal samples.
